# Mitochondria Superoxide Anion Production Contributes to Geranylgeraniol-Induced Death in *Leishmania amazonensis*

**DOI:** 10.1155/2012/298320

**Published:** 2012-12-04

**Authors:** Milene Valéria Lopes, Vânia Cristina Desoti, Angelo de Oliveira Caleare, Tânia Ueda-Nakamura, Sueli Oliveira Silva, Celso Vataru Nakamura

**Affiliations:** ^1^Programa de Pós-graduação em Ciências Farmacêuticas, Laboratório de Inovação Tecnológica no Desenvolvimento de Fármacos e Cosméticos, Bloco B-08, Universidade Estadual de Maringá, Avenida Colombo 5790, 87020-900 Maringá, PR, Brazil; ^2^Programa de Pós-graduação em Ciências Biológicas, Universidade Estadual de Maringá, Avenida Colombo 5790, 87020-900 Maringá, PR, Brazil

## Abstract

Here we demonstrate the activity of geranylgeraniol, the major bioactive constituent from seeds of *Bixa orellana*, against *Leishmania amazonensis*. Geranylgeraniol was identified through ^1^H and ^13^C nuclear magnetic resonance imaging and DEPT. The compound inhibited the promastigote and intracellular amastigote forms, with IC_50_ of 11 ± 1.0 and 17.5 ± 0.7 **μ**g/mL, respectively. This compound was also more toxic to parasites than to macrophages and did not cause lysis in human blood cells. Morphological and ultrastructural changes induced by geranylgeraniol were observed in the protozoan by electronic microscopy and included mainly mitochondria alterations and an abnormal chromatin condensation in the nucleus. These alterations were confirmed by Rh 123 and TUNEL assays. Additionally, geranylgeraniol induces an increase in superoxide anion production. Collectively, our *in vitro* studies indicate geranylgeraniol as a selective antileishmanial that appears to be mediated by apoptosis-like cell death.

## 1. Introduction

Leishmaniasis is a complex of diseases caused by protozoa of *Leishmania* genus, endemic in tropical and subtropical regions. Two million new cases of cutaneous and 0.5 million of visceral leishmaniasis are reported each year [1, 2]. The parasite's species are related to the clinical manifestations, which can vary from cutaneous lesions to a visceral dissemination. Localized cutaneous leishmaniasis is the most prevalent clinical manifestation. In Latin America, they are mainly caused by *Leishmania (Viannia) braziliensis* and *Leishmania (Leishmania) amazonensis* species [3]. 

Pentavalent antimonials, the first line of drugs currently used in the treatment of leishmaniasis, were developed several decades ago. These drugs are administrated by invasive routes and can cause serious side effects. Furthermore, they require long-term treatment and parasites have developed resistance. Thus, the development of new more effective and safer drugs continues to be the great challenge to cure leishmaniasis [4].

Natural compounds, known to be valuable sources of new medicinal agents, have been exhaustively evaluated against the trypanosomatids [5–8], in attempts to find the most effective compounds with better activities and fewer side effects. *Bixa orellana* L. (Bixaceae), known as “achiote” or “the annatto plant”, is a small evergreen tree native to rain forests of Central and South America. Its ethnomedical uses include treatment of constipation, fevers, heartburn, and asthma, and as a gastrointestinal tonic. The leaf extract is a good remedy for gonorrhea [9]. Previous studies have demonstrated that crude extracts from *B. orellana* show antimicrobial, antioxidant, antidiarrhoeal, anticonvulsant, platelet antiaggregant, and antifungal properties [10–12]. Furthermore, seed crude extract was found to be active against *L. amazonesis in vitro* [12].

Considering both the popular use of *B. orellana* in Brazilian medicine and the previous effect of seed crude extract against *L. amazonesis in vitro*, we investigated the potential effect of geranylgeraniol, a compound isolated from annatto seeds, in *L. amazonensis* and its possible targets in the parasite.

## 2. Materials and Methods

### 2.1. Plant Material

Annatto (*Bixa orellana* L.) seeds were collected, in the state of Paraná, Brazil. The taxonomic identity of the plant was confirmed, and a voucher specimen was deposited in the herbarium of the Department of Biology, State University of Maringá (HUM 11813).

### 2.2. Extraction, Concentration, and Identification of the Compound

Crude seeds of *Bixa orellana* (300 g) were extracted by Soxhlet apparatus under reflux with 500 mL of *n*-hexane for 8 h. After the extraction, the solvent was eliminated in a vacuum rotary evaporator at 40°C, to yield 6.7 g (2.2%) of an oil extract (OE) [13]. The OE was identified as geranylgeraniol by comparison of the NMR and DEPT spectra (Varian Gemini 300 (7.05 T) spectrometers) using deuterated solvent, TMS as the internal standard, and a constant temperature of 298 K. Low-resolution mass electrospray data were acquired in the positive ion mode using a Micro-119 mass Quattro-liquid chromatography instrument. Silica gel 60 (70–230 and 230–400 mesh); TLC: silica gel plates F254 (0.25–121 mm thickness). The HPLC (high-performance liquid chromatography) analyses were carried out using a Shimadzu apparatus LC-20T equipped with a pump LC-10AT, auto-sampler SIL-20A and a UV/VIS Photodiode Array Detector model SPD-M20A, controlled by a CBM-20A computer program. In the chromatographic analysis, we used a reverse-phase column Metasil ODS, 5 *μ*m, 150.0 × 4.6 mm, kept in an oven set at ambient temperature. HPLC conditions used acetonitrile/water (65:35, v/v) containing 2% acetic acid. The flow rate was 1 mL/min, and detection was at 450 nm. The reagents used to prepare the mobile phase were acetonitrile (HPLC grade from OmniSolv EM Science, Gibbstown, NJ), ultrapure water (Milli-Q system, Millipore, Bedford, USA), and acetic acid (analytical grade, Merck, Darmstadt, Germany). The OE solution of *Bixa orellana* seeds was prepared in acetonitrile/water (65:35, v/v) containing 2% acetic acid at a concentration of 1,000 *μ*g/mL. The solutions were filtered through a 0.45 *μ*m membrane filter (Millipore, SP, Brazil).

### 2.3. Preparation of Drugs

Geranylgeraniol and Amphotericin B (Cristalia Ltda, SP, Brazil) were diluted with DMSO and culture medium and the final solutions never exceeded 1% (v/v) and, at this concentration, the DMSO had no detectable effect on the parasites or mammalian cells used in the assays (data not shown). 

### 2.4. Parasite and Cell Culture

The MHOM/BR/75/Josefa strain of *L. amazonensis* was originally isolated from a patient with diffuse cutaneous leishmaniasis, by C. A. Cuba-Cuba (Universidade de Brasília, Brazil). Promastigote forms were cultured in Warren's medium (brain heart infusion plus haemin and folic acid) supplemented with 10% heat-inactivated fetal bovine serum (FBS) (Gibco Invitrogen Corporation, NY, USA) in a tissue flask at 25°C with weekly transfers. The J774G8 murine macrophages were cultured with RPMI 1640 medium (Gibco Invitrogen Co., Grand Island, New York, USA), with added sodium bicarbonate and L-glutamine, supplemented with 10% FBS, in tissue flasks, at 37°C in a 5% CO_2_-air mixture [14].

### 2.5. Antileishmanial Activity

 Promastigote forms of the parasite in the logarithmic-phase (1 × 10^6^ parasites/mL) were cultured on a 24-well plate in Warren's medium supplemented with FBS in the absence or in the presence of 10, 20, 30, 40, 50, or 100 *μ*g/mL of geranylgeraniol. The activity against promastigotes was evaluated after 24, 48, 72, and 96 h of incubation. The results were expressed as log number cells/mL, and the IC_50_ (50% inhibitory concentration) was determined at 48 h post incubation [15]. Amphotericin B was used as the positive control. 

Afterwards, based in the antipromastigote effect of geranylgeraniol, we evaluate the anti-amastigote intracellular activity of the compound in concentrations of 1 to 25 *μ*g/mL. For this, peritoneal resident cells (1 × 10^5^ cells/mL) from BALB/c male mice were harvested in RPMI 1640 medium supplemented with 10% FBS and 40 mg/mL gentamicin. The cells were plated on coverslips (13 mm diameter) in 24-well plates and incubated for 16 h. Next, adhered macrophages were infected with 10 promastigotes per host cell and incubated for 6 h at 37°C in 5% CO_2_ atmosphere. Geranylgeraniol at 1, 5, 10, or 25 *μ*g/mL was added to infected macrophages and after 24 h the coverslips were fixed in methanol and stained with Giemsa. The number of amastigotes was determined by counting at least 200 macrophages, and the results were expressed as the survival index (multiplying of infected macrophage percentage by the mean number of internalized parasites per cell) [15]. Amphotericin B and nontreated infected macrophages were used as positive and negative control, respectively. 

### 2.6. Cytotoxicity Assay in Macrophage Cells

The cytotoxicity was evaluated in J774G8 macrophage cells. A suspension of 5 × 10^5^ cells/mL was cultured in RPMI 1640 medium supplemented with 10% FBS and added to each well in 96-well microplates. The plates were incubated at 37°C in a 5% CO_2_-air mixture to obtain confluent growth of the cells. After 24 h, the compound was added to each well in crescent concentrations starting from IC_50_ of geranylgeraniol (10, 50, 100, 500, or 1,000 *μ*g/mL), and the plates were incubated for 48 h in a 5% CO_2_-air mixture at 37°C. Then, the cultures were fixed with 10% trichloroacetic acid at 4°C for 1 h and stained in 0.4% sulforhodamine B (SRB) in 1% acetic acid for 30 min at 4°C. The microplate was washed four times with 1% acetic acid, and 150 *μ*L/well of 10 mM unbuffered trisbase solution (Sigma Chemical Co, MO, USA) was added and then homogenized [16]. Absorbance was read in a 96-well plate reader (BIO-TEK Power Wave XS) at 530 nm. The percentage of viable cells was calculated in relation to controls consisting of cells cultured in medium alone, by CC_50_ values (50% cytotoxicity concentration). The CC_50_ was determined by logarithm regression analysis. Amphotericin B was used as positive control of cytotoxicity.

### 2.7. Hemolytic Assay

Healthy human type A erythrocytes were defibrinated, and a 3% solution in 0.85% glycosylated saline was prepared. The solution was incubated with different concentrations of geranylgeraniol (5, 10, 25, 50, 100, 250, or 500 *μ*g/mL), in a microplate at 37°C. After 2 h, the microplate was centrifuged and the hemoglobin released was determined in the supernatant by absorbance in an ELISA reader at 550 nm. The positive and negative controls used for comparison were Triton X-100 and an erythrocyte suspension, respectively. Amphotericin B was the reference drug utilized. The results were expressed as the percentage of hemolysis calculated in regard to Amphotericin B [17]. 

### 2.8. Scanning Electron Microscopy

Promastigote forms (1 × 10^6^ parasites/mL) in the absence or in the presence of 11 *μ*g/mL of geranylgeraniol for 48 h were washed with 0.01 M PBS and fixed in 2.5% glutaraldehyde in 0.1 M sodium cacodylate buffer at 4°C for 48 h. The parasites were adhered on the poly-L-lysine-coated coverslip, dehydrated in different concentrations of ethanol, critical point-dried with CO_2_, sputter-coated with gold, and observed in a Shimadzu SS-550 scanning electron microscope [18].

### 2.9. Transmission Electron Microscopy

Promastigote forms (1 × 10^6^ parasites/mL) in the absence or in the presence of 11 *μ*g/mL of geranylgeraniol for 48 h were harvested by centrifugation and fixed in 2.5% glutaraldehyde in 0.1 M sodium cacodylate buffer. Next, the cells were post-fixed in a solution containing 1% osmium tetroxide and 0.8% potassium ferrocyanide at room temperature for 60 min, dehydrated in different concentrations of acetone, and embedded in Epon resin. Thin sections were stained with uranyl acetate and lead citrate, to be examined in a Zeiss 900 transmission electron microscope [14].

### 2.10. Mitochondria Membrane Potential and Cell Membrane Integrity Assay

Promastigote forms (5 × 10^6^ parasites/mL) in the absence or in the presence of 100 *μ*g/mL of geranylgeraniol for 3 h were harvested and washed with PBS. Then, the parasites were washed and incubated at 37°C with rhodamine 123 (Rh 123) (5 *μ*g/mL for 15 min) to evaluate the mitochondria membrane potential (ΔΨ_m_), and with propidium iodide (PI) (0.2 *μ*g/mL for 10 min) to verify possible alteration in cell membrane integrity. The compound carbonyl cyanide *m*-chlorophenylhydrazone (CCCP) (100 *μ*M) and amphotericin B (5 *μ*M) were used as positive control for mitochondria membrane potential alteration and cell membrane alteration, respectively. The material was kept on ice until analysis. The mean of fluorescence intensity of the cells was analyzed by flow cytometry FACSCalibur and CellQuest software. A total of 10,000 events were acquired in the region previously established as that corresponding to the parasites [19].

### 2.11. Fluorimetric Detection of Mitochondria-Derived O_2_^∙−^

Promastigote forms (2 × 10^7^ parasites/mL) were harvested and washed with Krebs-Henseleit (KH) solution buffer, that contained 15 mM NaHCO_3_, 5 mM KCl, 120 mM NaCl, and 0.7 and 1.5 mM NaH_2_PO_4_ (pH 7.3). The cells were loaded with 5 *μ*M MitoSOX reagent [3,8-phenanthridinediamine, 5-(6-triphenylphosphoniumhexyl)-5,6-dihydro-6-phenyl; Invitrogen)] (Molecular Probes, Eugene, OR, USA). The parasites were incubated for 10 min at room temperature (22°C) and protected from light. After incubation with MitoSOX reagent, the parasites were washed two times with KH buffer and untreated or treated with 11 *μ*g/mL and 30 *μ*g/mL. Antimycin A at 10 *μ*M, a stimulus known to induce superoxide anion (O_2_^∙−^) production by mitochondria, was used as positive control. MitoSOX detection was performed using black 96-well plates for 2 h. Fluorescence was measured in a fluorescence microplate reader (Victor X3 - PerkinElmer) at *λ*_ex_ = 510 nm and *λ*_em_ = 580 nm [20]. The results are expressed as arbitrary units of MitoSOX.

### 2.12. DNA Fragmentation Assay

DNA double-strand ruptures were analyzed* in situ* by TUNEL assay (Terminal Deoxynucleotide Transferase dUTP Nick End Labeling). For this, promastigote forms (1 × 10^6^ parasites/mL) were incubated in the absence or in the presence of geranylgeraniol at 11 *μ*g/mL for 48 h. The parasites were fixed with paraformaldehyde 1% and subjected to the TUNEL assay as recommended by the manufacturer (Molecular Probes, Eugene, OR, USA). Parasites that have undergone DNA double-strand ruptures should fluoresce brightly, unlike the untreated parasites. Fluorescence was observed in a fluorescence microscope Olympus BX51 (Olympus) and pictures were captured with a UC30 camera (Olympus). 

### 2.13. Statistical Analysis

Statistical analyses were performed using the program GraphPad Prism 4 (GraphPad Software, San Diego, CA, USA). The data shown in the graph was expressed as means ± standard deviation of the mean of independent experiments. *P* ≤ 0.05 was adopted as the minimum criterion of significance. Data were analyzed with one-way analysis of variance (ANOVA) and significant differences among means were identified with post hoc Tukey testing. 

## 3. Results

### 3.1. Identification of Geranylgeraniol

The compound obtained from the seeds of* Bixa orellana *was identified as geranylgeraniol by ^1^H-NMR, ^13^C-NMR and DEPT analyses and the data were compared to data from literature [21]. Electrospray ionization mass spectrometry, m/z (relative intensity): 291 [M+H]^+^ (100); ^1^H nuclear magnetic resonance (NMR) (CDCI_3_, tetramethylsilane (TMS) internal standard) **δ** (ppm), 3.72 (2H, dd, *J* = 14.1, and 6.9 Hz, H-1), 5.09-5.11 (4H, m, H-2, H-6, H-10, and H-14), 1.99-2.07 (10H, m, H-4, H-5, H-8, H-9, and H-12), 1.60 (6H, s, H-4′, and H-8′), 1.68 (6H, s, H-12′, and H-16), 1.24 (3H, s, H-16′), and 0.94 (1H, t, *J* = 6.9 Hz, 1-OH); ^13^C NMR (CDCI_3_, TMS internal standard) **δ** (ppm), 16.19 (C-8′), 16.47 (C-12′), 18.53 (C-16′), 17.87 (C-4′), 26.50 (C-9), 25.89 (C-16), 26.80 (C-13), 26.94 (C-5), 39.75 (C-4), 39.87 (C-8), 39.90 (C-12), 59.52 (C-1), 123.39 (C-2), 123.90 (C-6), 124.35 (C-10), 124.57 (C-14), 131.50 (C-15), 135.18 (C-7), 135.32 (C-11), and 140.09 (C-3). 

### 3.2. Antileishmanial Activity

**Figure 1:**
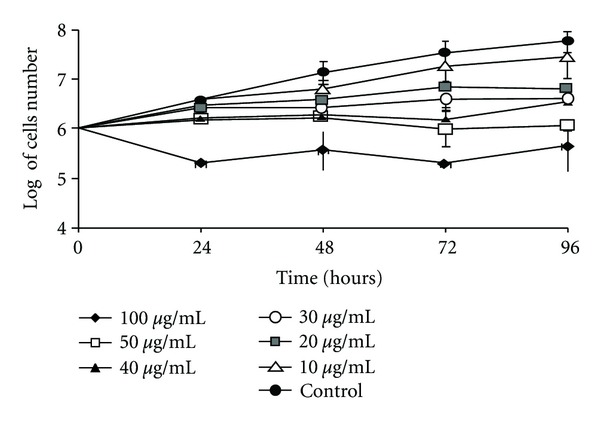
Effects of geranylgeraniol on promastigote form of *Leishmania amazonensis* treated for 96 h. These results were from two experiments in duplicate and were expressed as log of parasite number ± standard deviations of growth inhibition in relation to untreated protozoa.

Geranylgeraniol obtained from the seeds of *B. orellana* was tested initially against the promastigote form of *L. amazonensis. *The results indicated a progressive inhibition in a dependent concentration growth (Table 1). The IC_50_ value after 48 h of incubation was 11 ± 1.0 *μ*g/mL (38 *μ*M) (Figure 1).

**Figure 2:**
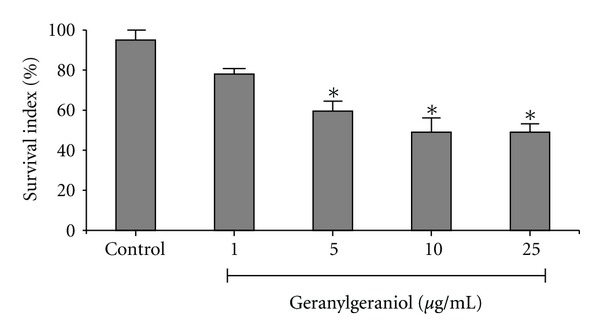
Effect of geranylgeraniol on macrophages interaction with *Leishmania amazonensis* after 24 h of treatment. Peritoneal macrophage cells were infected with promastigotes and treated with 1, 5, 10, or 25 *μ*g/mL. Control group (untreated parasites) is also shown. The survival index was calculated by multiplying the percentage of macrophage cells with parasites and the mean number of internalized parasites per cell. The significant results (*P < 0.05) represent data from two independent experiments.

The effect of geranylgeraniol on intracellular amastigotes was observed after 24 h of incubation (Figure 2). The survival indexes were 78, 59.5, 49 and 49% for 1, 5, 10 and 25 *μ*g/mL of the drug, respectively. Survival indexes of treated amastigotes were significantly (*P* < 0.05) different from that of non-treated macrophages. The compound showed an IC_50_ value of 17.5 ± 0.7 *μ*g/mL (60 *μ*M).

The IC_50_ for the positive control, amphotericin B, was 0.058 *μ*g/mL (0.06 *μ*M) and 0.26 *μ*g/mL (0.3 *μ*M) against the promastigotes and amastigotes, respectively (data not shown).

### 3.3. Cytotoxicity Assay

This assay evaluated the potential toxic effects of geranylgeraniol on the J774G8 murine macrophages, after 48 h of treatment. When macrophages were treated with geranylgeraniol, the 50% cytotoxic concentration was 41.5 ± 3.5 *μ*g/mL (143 *μ*M) (data not shown). The toxicity for J774G8 macrophages was compared with the activity against the promastigote form, obtaining the selectivity index (SI) (CC_50_ for J774G8 cells/IC_50_ for protozoa). Geranylgeraniol was more selective against the promastigotes than the mammalian cells, with an SI ratio of 3.8.

### 3.4. Hemolytic Assay

The hemolytic potentials of geranylgeraniol and the reference drug Amphotericin B were tested and compared with positive (Triton X-100) and negative control (erythrocyte suspension). After 2 h of incubation at 37°C, the results indicated a significantly low hemolysis percentage (*P* < 0.05) of 26.4% ± 6.5 for geranylgeraniol at 500 *μ*g/mL (data not shown). In contrast, the reference drug showed a hemolytic effect of 81.9% ± 7.7 at the same concentration. 

### 3.5. Scanning Electron Microscopy

**Figure 3:**
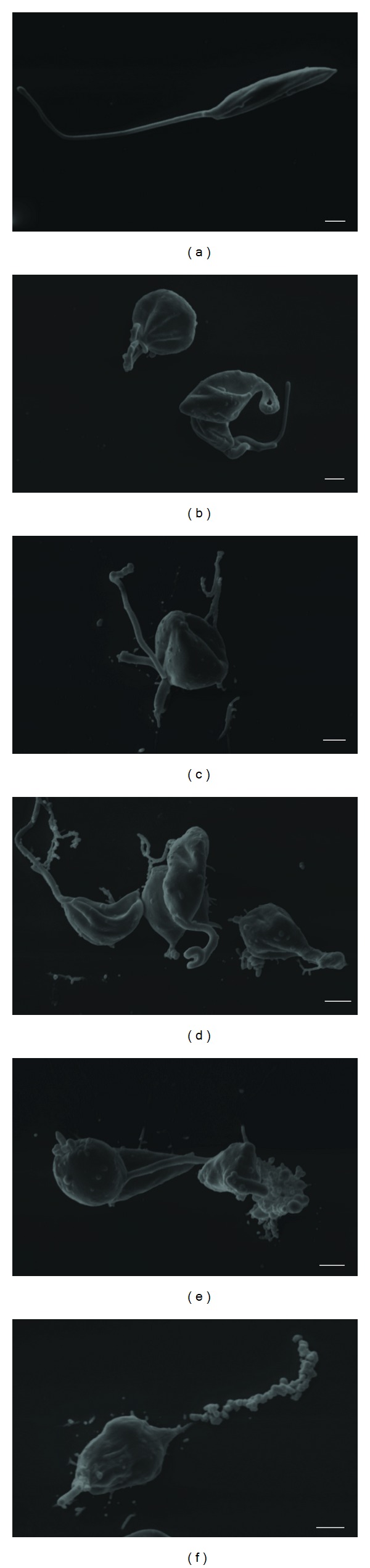
Scanning electron microscopy of promastigote forms of *Leishmania amazonensis *after 48 h of treatment with geranylgeraniol. (a) Control (untreated parasites); (b)–(f) parasites treated with geranylgeraniol at 11 *μ*g/mL. Bars = 1 *μ*m.

Morphological alterations of *L. amazonensis* treated with geranylgeraniol were observed by scanning electron microscopy. The compound at 11 *μ*g/mL caused rounding and swelling of the parasite (Figures 3(b) and 3(c)). The protozoa also showed rupture of the plasma membrane, cell lysing, and significant alterations of the flagellar membrane (Figures 3(d), 3(e) and 3(f)).  Figure 3 shows the characteristic elongated shape of an untreated protozoan with a terminal flagellum.

### 3.6. Transmission Electron Microscopy

**Figure 4:**
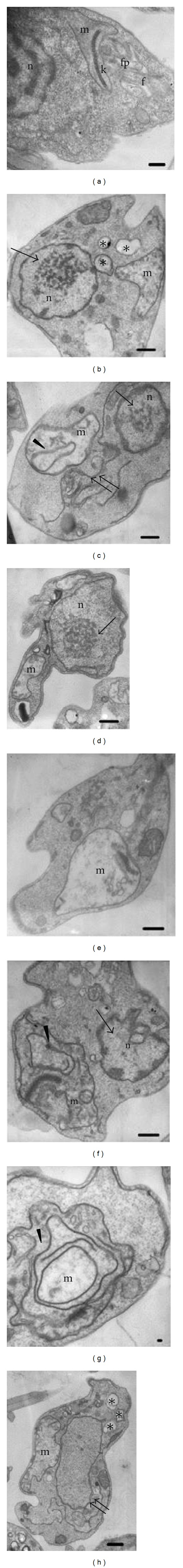
Transmission electron microscopy of *Leishmania amazonensis* promastigotes after 48 h of treatment with geranylgeraniol. (a) Untreated promastigotes showing normal mitochondria (m), nucleus (n), typical kinetoplast (k), flagellum (f), and flagellar pocket (fp); (b)–(h) promastigote treated with geranylgeraniol at 11 *μ*g/mL. The compound led to cytoplasmic vacuolization (black asterisk), presence of concentric membranes inside mitochondria (arrowhead), abnormal chromatin condensation in nucleus (arrow), and an endoplasmic reticulum profile surrounding cytoplasmic structures (double arrows). Bars = 1 *μ*m.

Ultrastructural analysis of the promastigote form treated with geranylgeraniol revealed significant alterations. Mitochondria showed intense swelling, the presence of concentric membrane structures inside the organelle, and a less electron-dense matrix (Figures 4(c), 4(f), and 4(g)). The parasites showed alterations in the nucleus, which became enlarged and with an irregular surface, and an abnormal chromatin condensation with DNA fragmentation (Figures 4(b), 4(c), 4(d) and 4(f)). The presence of cytoplasmic vacuoles (Figures 4(b) and 4(h)) and endoplasmic reticulum surrounding cytoplasmic structures and organelles (Figures 4(c) and 4(h)) was also observed. These ultrastructural changes were not observed in untreated parasites, which had cytoplasm with a dense matrix and regular membranes (Figure 4(a)).

### 3.7. Effects of Geranylgeraniol on Mitochondria Membrane Potential and Plasma Membrane Integrity

**Figure 5:**
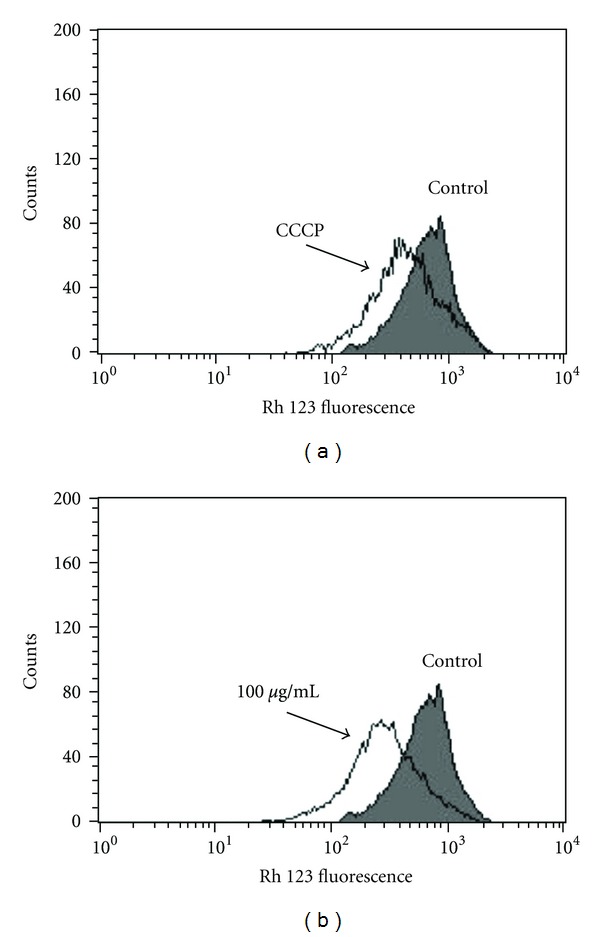
Flow cytometry analysis of *L. amazonensis* treated with geranylgeraniol for 3 h and labeled with Rh 123. (a) Promastigotes treated with 100 *μ*M of CCCP (positive control). (b) Promastigotes treated with 100 *μ*g/mL of geranylgeraniol. Control group (untreated parasites) was also showed. Typical histogram of at least three independent experiments.

To confirm the effect of geranylgeraniol in parasite's mitochondria, we decided to evaluate the ΔΨm in treated promastigotes after 3 h of incubation with 100 *μ*g/mL, using flow cytometry. The treatment of promastigotes caused a decrease in Rh 123 total fluorescence intensity of 50.2%, when compared to the control group, indicating mitochondria depolarization (Figure 5(b)). Furthermore, a depolarization in mitochondria membrane potential values was also observed following treatment with CCCP (26.4%) (Figure 5(a)). However, the treatment does not affect the plasma membrane integrity (by PI labeling) (data not shown). 

### 3.8. Detection of Mitochondria-Derived O_2_^∙−^ of Promastigote Forms

**Figure 6:**
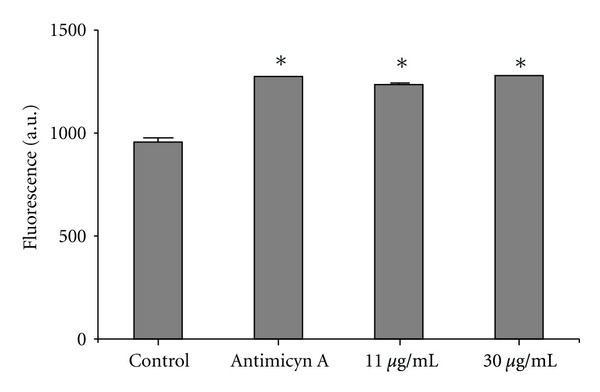
Mitochondria O_2_^∙−^ production in *Leishmania amazonensis* promastigotes treated with geranylgeraniol for 2 h. Mitochondria O_2_^∙−^ production was evaluated using the fluorescent probe MitoSOX. Asterisks indicate significant difference relative to the control group (untreated parasites) as identified by variance analysis (one-way) with post hoc Tukey testing (*P ≤ 0.05). Results are expressed as mean fluorescence (in arbitrary units) ± SD of at least three independent experiments.

As shown in Figure 6, geranylgeraniol induced a significantly increase in the O_2_^∙−^ production in both concentrations assayed even at low concentration (11 and 30 *μ*g/mL) with 2 h of incubation, when compared to the control group. The positive control with AA also induced an increase of mitochondria O_2_^∙−^ production.

### 3.9. Effect of Geranylgeraniol on DNA Fragmentation of Promastigote Forms

**Figure 7:**
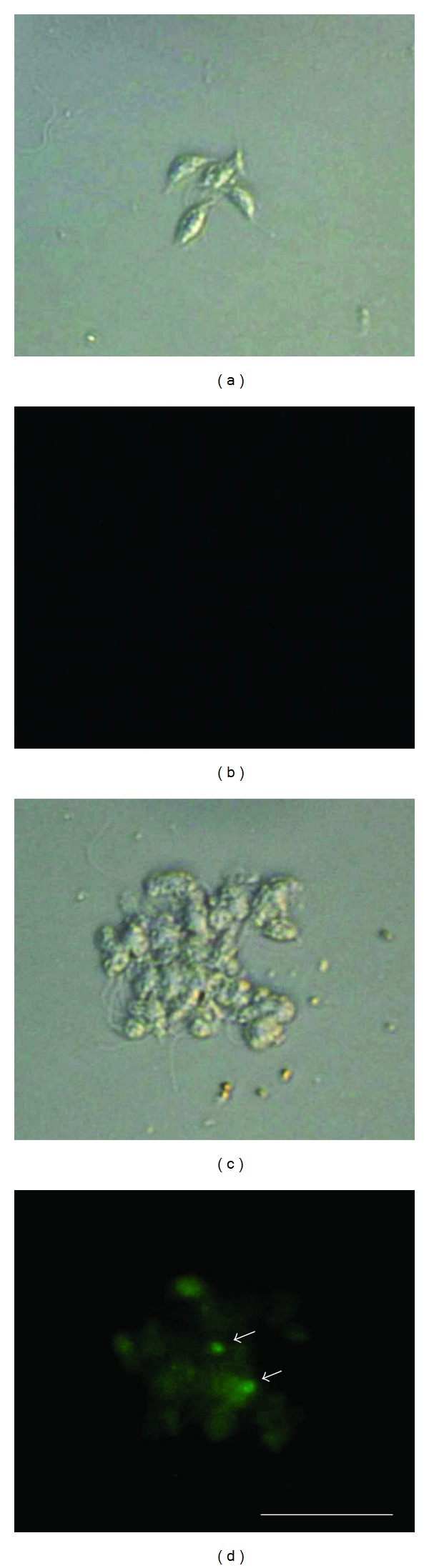
Determination of DNA fragmentation in *Leishmania amazonensis* promastigotes treated with geranylgeraniol for 48 h. Gray column is differential interference contrast (DIC) and black column is fluorescence. (a), (b) Representative images of untreated parasites. (c), (d) Representative images of promastigotes treated with 11 *μ*g/mL. Arrows indicate DNA fragmentation (green). Bars = 20 *μ*m.

The DNA fragmentation experiment, documented through the DNA labelling, confirmed the electron microscopy results in geranylgeraniol-treated promastigotes. As shown in Figure 7, the fluorescence was bright in parasites treated with 11 *μ*g/mL of geranylgeraniol for 48 h (d) when compared to the control group (untreated parasites) (b).

## 4. Discussion

In a previous study, a methanol extract from the seeds of *B. orellana* showed antileishmanial activity against *L. amazonensis* and* L. chagasi* with an IC_50_ value of 22 and 250 *μ*g/mL, respectively [12]. In the present study, we evaluated the pharmacological activity of geranylgeraniol obtained from *B. orellana* seeds, against *L. amazonensis*,* in vitro*. Geranylgeraniol was identified by NMR and DEPT analyses and the data agree with those reported by Coates et al. [21]. This compound has also previously been isolated and characterized from *Pterodon pubescens* seeds and fruit oil and *Croton lobatus* leaves [22–24]. Previous quantitative studies demonstrated that the hexane extract from the seeds of *B. orellana* contains 57% of geranylgeraniol. Furthermore, the farnesyalcetate was the second compound identified in the hexane extract and it seems that it is a product from geranylgeraniol degradation [13]. Additionally, geranylgeraniol was the sole compound obtained by HPLC from the hexane fraction of *Pterodon pubescens* [24]. 

Geranylgeraniol showed alterations in mitochondrial membrane potential (ΔΨm) and fragmentation of DNA of *L. amazonensis* and these data are very similar to the activity of geranylgeraniol on *T. cruzi* [24]. Additionally, our results indicated that geranylgeraniol was not toxic to the human erythrocytes even at high concentrations and was more selective to the parasite than to human cells. 

Based on the results described above, we further investigated the leishmanicidal mechanism of geranylgeraniol. We observed, by electron microscopy, drastic ultrastructural alterations especially in the mitochondria and in the nuclei of the treated parasites. In fact, the mitochondria of Trypanosomatids exhibit unique characteristics that are distinct from mammalian mitochondria, making this organelle a major target of chemotherapeutic agents [25]. Additionally, increasingly more papers have been published that describe leishmanicidal compounds that target *Leishmania *mitochondria [26–28]. These alterations were confirmed by Rh 123-labeled and TUNEL assays. These assays are highly sensitive and demonstrated alterations in ΔΨm and fragmentation of DNA. Additionally, our results also indicated that geranylgeraniol stimulate O_2_^∙−^ production in mitochondria, by MitoSOX assay, similar to the effect of a compound studied by Britta et al. [27]. 

The experiments presented herein showed that geranylgeraniol increased O_2_^∙−^ production as soon as treatment began. In contrast, geranylgeraniol induced mitochondrial membrane depolarization after 3 h treatment and only with higher concentration, which was observed in the Rh 123 assay. Our overall hypothesis is that the mechanism of action of geranylgeraniol involves an increase in reactive oxygen species (ROS) that acts in any membrane of the parasite, including the mitochondrial membrane leading to membrane permeabilization followed by mitochondrial depolarization and increase in mitochondrial ROS production through the electron transport chain. Up to here, we observed that geranylgeraniol is able to induce oxidative imbalance, a disorder induced also by promising leishmanicidal compounds [28, 29]. In such a situation, oxidative damage is expected and DNA is one of the biological macromolecules that can undergo destructive effects.

Our results suggest that the type of alterations induced by geranylgeraniol, isolated from the seeds of *B. orellana*, in *L. amazonensis* promastigotes is typical of cells undergoing apoptosis-like death [30]. Apoptosis is an active process of cell death and has important roles in maintaining homeostasis in multicellular organisms. It is defined in terms of characteristic morphological changes including reduction in cell volume, condensation of the chromatin, fragmentation of DNA, and preservation of the plasma membrane [30].

The literature provides a number of examples that protozoa parasites such as *Leishmania* can undergo programmed cell death (PCD) in response to stress and drug application [31–34]. These examples suggest PCD based on depolarization of the mitochondrial membrane potential and fragmentation of nuclear DNA [32]. Geranylgeraniol has demonstrated a strong apoptosis-inducing activity in several lines of human cancer cells [35, 36]. Promastigotes of *L. donovani*, after treatment with diospyrin and ethanolamide, exhibited depolarization of the ΔΨ_m_ and fragmentation of nuclear DNA, characteristics typical of PCD, indicating the initiation of “apoptosis-like death” in the promastigotes [33]. This effect was also observed after treatment with camptothecin, miltefosine, and amphotericin B [31, 32, 34]. Therefore, it appears that geranylgeraniol could be explored for the development of a new antileishmanial drug. 

## Figures and Tables

**Table 1:** IC_50_ values of *Leishmania amazonensis* promastigotes after treatment with geranylgeraniol in different times of incubation.

Time post incubation (h)	IC_50 _(*μ*g/mL)^a^	CI 95%^b^	*R* ^2^
24	37 ± 14.1	35.4 to 48.4	0.90
48	11 ± 1.0	9.2 to 18.3	0.87
72	10.5 ± 0.7	9.9 to 14.0	0.96
96	11.5 ± 2.1	8.8 to 12.8	0.95

^a^Values are representative of two independent experiments.

^b^95% confidence interval.
